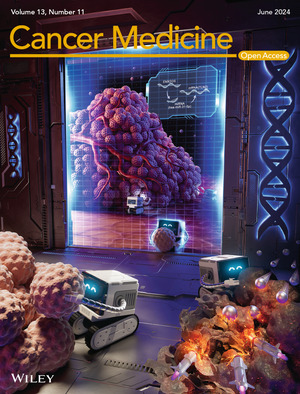# Cover Image

**DOI:** 10.1002/cam4.7437

**Published:** 2024-06-28

**Authors:** Zhan Yang, Xiaoting Zhang, Ning Zhan, Lining Lin, Jingyu Zhang, Lianjie Peng, Tao Qiu, Yaxian Luo, Chundi Liu, Chaoran Pan, Junhao Hu, Yifan Ye, Zilong Jiang, Xinyu Liu, Mouyuan Sun, Yan Zhang

## Abstract

The cover image is based on the Research Article *Exosome‐related lncRNA score: A value‐based individual treatment strategy for predicting the response to immunotherapy in clear cell renal cell carcinoma* by Zhan Yang et al., https://doi.org/10.1002/cam4.7308.